# Hepatic macrophage niche: a bridge between HBV-mediated metabolic changes with intrahepatic inflammation

**DOI:** 10.3389/fimmu.2024.1414594

**Published:** 2024-07-18

**Authors:** Jun Wang, Hongzhou Lu, Qian Li

**Affiliations:** ^1^ The Third People’s Hospital of Shenzhen (National Clinical Research Center for Infectious Diseases) and The Second Affiliated Hospital of Southern University of Science and Technology, Shenzhen, Guangdong, China; ^2^ Clinical Research Center, The Fifth People’s Hospital of Wuxi, Jiangnan University, Wuxi, Jiangsu, China

**Keywords:** HBV, hepatic macrophage niches, liver inflammation, metabolism, lipid metabolism (fatty acids)

## Abstract

Hepatitis B Virus (HBV) is a stealthy and insidious pathogen capable of inducing chronic necro-inflammatory liver disease and hepatocellular carcinoma (HCC), resulting in over one million deaths worldwide per year. The traditional understanding of Chronic Hepatitis B (CHB) progression has focused on the complex interplay among ongoing virus replication, aberrant immune responses, and liver pathogenesis. However, the dynamic progression and crucial factors involved in the transition from HBV infection to immune activation and intrahepatic inflammation remain elusive. Recent insights have illuminated HBV’s exploitation of the sodium taurocholate co-transporting polypeptide (NTCP) and manipulation of the cholesterol transport system shared between macrophages and hepatocytes for viral entry. These discoveries deepen our understanding of HBV as a virus that hijacks hepatocyte metabolism. Moreover, hepatic niche macrophages exhibit significant phenotypic and functional diversity, zonal characteristics, and play essential roles, either in maintaining liver homeostasis or contributing to the pathogenesis of chronic liver diseases. Therefore, we underscore recent revelations concerning the importance of hepatic niche macrophages in the context of viral hepatitis. This review particularly emphasizes the significant role of HBV-induced metabolic changes in hepatic macrophages as a key factor in the transition from viral infection to immune activation, ultimately culminating in liver inflammation. These metabolic alterations in hepatic macrophages offer promising targets for therapeutic interventions and serve as valuable early warning indicators, shedding light on the disease progression.

## 1 Introduction

Chronic Hepatitis B (CHB) affects metabolic processes, potentially contributing to the development of Metabolic Dysfunction-Associated Fatty Liver Disease (MASLD). This dual pathology intensifies further liver injury, increasing the risks of cirrhosis and liver cancer, and imposing a significant disease burden (1–4). Although CHB and MASLD share common risk factors for liver fibrosis and cirrhosis, particularly host metabolic factors, the causal link between hepatitis B virus (HBV) infection and MASLD remains elusive (1, 2). This is largely due to an incomplete understanding of how HBV impacts host metabolic processes and subsequently influences immunopathogenesis.

The inflammatory responses induced by the HBV are capable to exacerbate hepatic steatosis through various mechanisms. HBV DNA transcription can lead to metabolic reprogramming in the liver, accelerating processes such as hepatic regeneration, inflammation, and fibrosis (5). The HBV X (HBx) protein activates signaling pathways such as Phosphoinositide 3-kinase (PI3K)/protein kinase B (AKT) and Toll-like receptors (TLRs), which influence hepatic lipogenesis, the conversion of cholesterol to bile acids (BAs), and hepatic lipid homeostasis, thereby contributing to hepatic steatosis (6–9). Additionally, HBV pre-S1 binding to NTCP) may alter hepatic cholesterol metabolism, leading to hepatic steatosis (10). Despite these findings collectively support the notion that metabolism plays a significant role in liver inflammation in CHB, the specific details of the intermediate mechanisms connecting intrahepatic metabolic change with immune infiltration during CHB remain unclear.

Growing evidence has reinforced the notion that HBV can manipulate metabolic processes by cross-talking with macrophages. Basically, HBV was known as a stealthy virus which can hijack the host’s BAs metabolic pathway. This can occur either by directly sequestering them through binding to the NTCP receptor or by being taken up by a subtype of macrophages via the cholesterol transport system to hepatocytes for viral entry (10). Consequently, the overloaded lipid transport system leads to compensatory up-regulation of BAs and dysfunctional cholesterol metabolism, resulting in the accumulation of lipid metabolites. This accumulation gradually triggers an intensified inflammatory response within hepatic macrophages (11, 12). With consistent exposure to lipid metabolites, hepatic macrophages are likely to undergo significant phenotypic and functional changes, primarily within the vicinity of the liver portal area, bile ducts, or lipid-accumulating regions (13, 14). For instance, a specific subset of liver macrophages, Trem2+ macrophages, has been found to shift toward a pro-inflammatory state in CHB-ACLF (14). Additionally, hepatic macrophages can release chemokines such as C-X-C motif chemokine ligand 9 and 10 (CXCL9 and CXCL10), which attract lymphocytes and monocytes/macrophages, thereby further contributing to liver inflammation (15). Hence, liver macrophages act as a conduit and potentially connect HBV-induced metabolic shifts with immune infiltrates exhibiting zonation characteristics.

The current consensus recognizes that hepatic niche macrophages, comprising resident macrophages, Kupffer cells (KCs), and monocyte-derived macrophages (MDMs) and their various subsets, play a crucial role in maintaining hepatic homeostasis by participating in various processes, including metabolic regulation, modulation of inflammation, promotion of immune tolerance, support for immunologic cell death, and facilitation of tissue repair in the physiological state (16–18). Despite researchers having a profound understanding of these issues, discussions regarding the involvement of hepatic macrophages in CHB progression have been still prominent, particularly in relation to three questions: 1) whether they promote immune activation or immune tolerance during CHB, 2) how they perceive hepatocyte stress or directly detect pathogens, and 3) what are their intrahepatic roles in HBV infection, metabolic hijacking and immune inflammation?

This review provides a contemporary insight into the knowledge accumulated in recent years. We emphasize the novel perspectives for intrahepatic metabolic alterations during HBV entry and replication, and shedding light on the multifaceted functions of hepatic macrophages during CHB processes, including bridge between HBV-Mediated Metabolic Change with Intrahepatic Inflammation.

## 2 Metabolic changes and characteristics during HBV entry and intrahepatic replication

### 2.1 Novel route for HBV entry: macrophage-mediated reverse cholesterol transport enhances HBV targeting in hepatocytes

**Figure 1:**
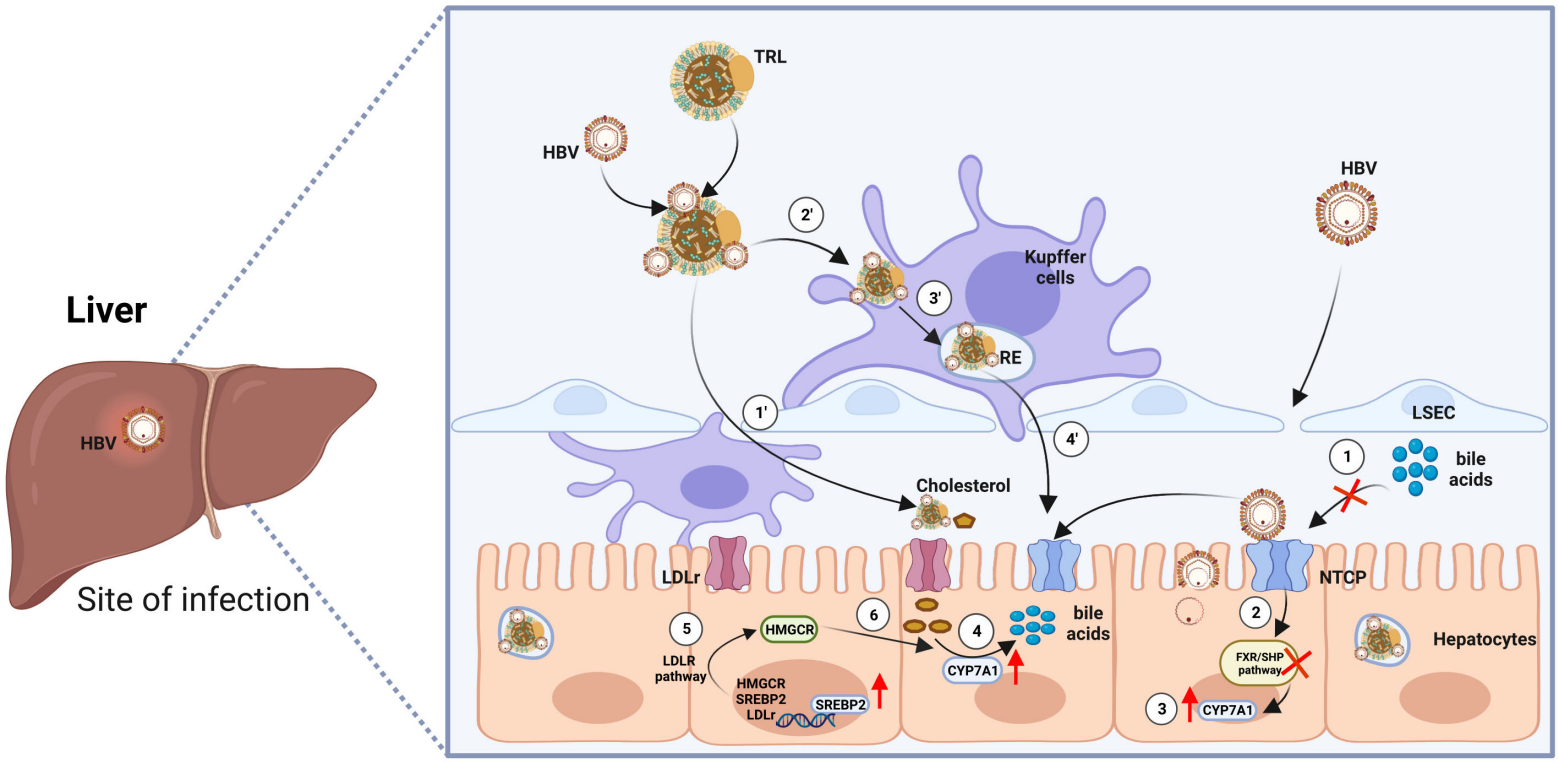
Model of HBV entry into hepatocytes via two routes. First Route: ① Enveloped hepatitis B virus (HBV) particles circulate within the sinusoidal blood, traversing the fenestrae of sinusoidal endothelial cells to access the space of Disse. Eventually, they reach the integral membrane protein receptor NTCP, which concurrently impedes the transport of bile acids. ② Repressed bile acid transport inhibits the farnesoid X receptor/small heterodimer partner (FXR/SHP) signaling pathway, leading to the compulsory upregulation of the transcription factor Cytochrome P450 Family 7 Subfamily A Member 1 (CYP7A1) ③. ④ Increased CYP7A1 accelerates the reversed transport of cholesterol and promotes the synthesis of bile acids. ⑤ The low density lipoprotein receptor (LDLR) pathway is activated, further promoting the process of bile acid synthesis. Second Route: ① HBV became entrapped within serum triglyceride-rich lipoproteins (TRL), traverse the endothelial cell layer to access the space of Disse. Once there, they are ensnared through electrostatic interactions with HSPG (heparan sulfate proteoglycan) before ultimately binding with NTCP (sodium taurocholate co-transporting polypeptide). ② ③ When associated with lipoproteins, HBV is preferentially internalized by liver macrophages into recycling endosomes. ④ Subsequently, HBV particles enter hepatocytes. Cholesterol esters of endocytosed lipoproteins undergo hydrolysis by the endosomal acid lipase, liberating free cholesterol. This free cholesterol is then conveyed to the cell membrane, released from the macrophage, and can bind to extracellular lipid receptors, facilitating additional uptake into hepatocytes. The figure was created by BioRender (BioRender.com).

Although HBV is known to enter hepatocytes through NTCP, the mechanism by which a single HBV particle can establish chronic liver infection after intravenous injection, evading scavenger cells in the circulation or spleen, has remained a puzzle (10, 19). Surprisingly, Esser K. et al., utilizing an ex vivo liver perfusion model, discovered that HBV, in association with lipoproteins, exhibited a tendency to be taken up by liver macrophages, leading to its accumulation in recycling endosomes before being re-secreted by macrophages, this time in association with free cholesterol obtained from the endocytosed lipoproteins. Subsequently, in the same ex vivo model, HBV was taken up by hepatocytes along with macrophage-derived neutral lipids (20). These findings support the conclusion that HBV efficiently targets hepatocytes not solely through a routine viral-specific host cell approach but also by exploiting neighboring cells in the liver. This research provides compelling evidence that HBV associates with lipoproteins, using the cholesterol transport pathway through macrophages and their cargo delivery system to reach hepatocytes (**Figure 1**).

**Figure 2:**
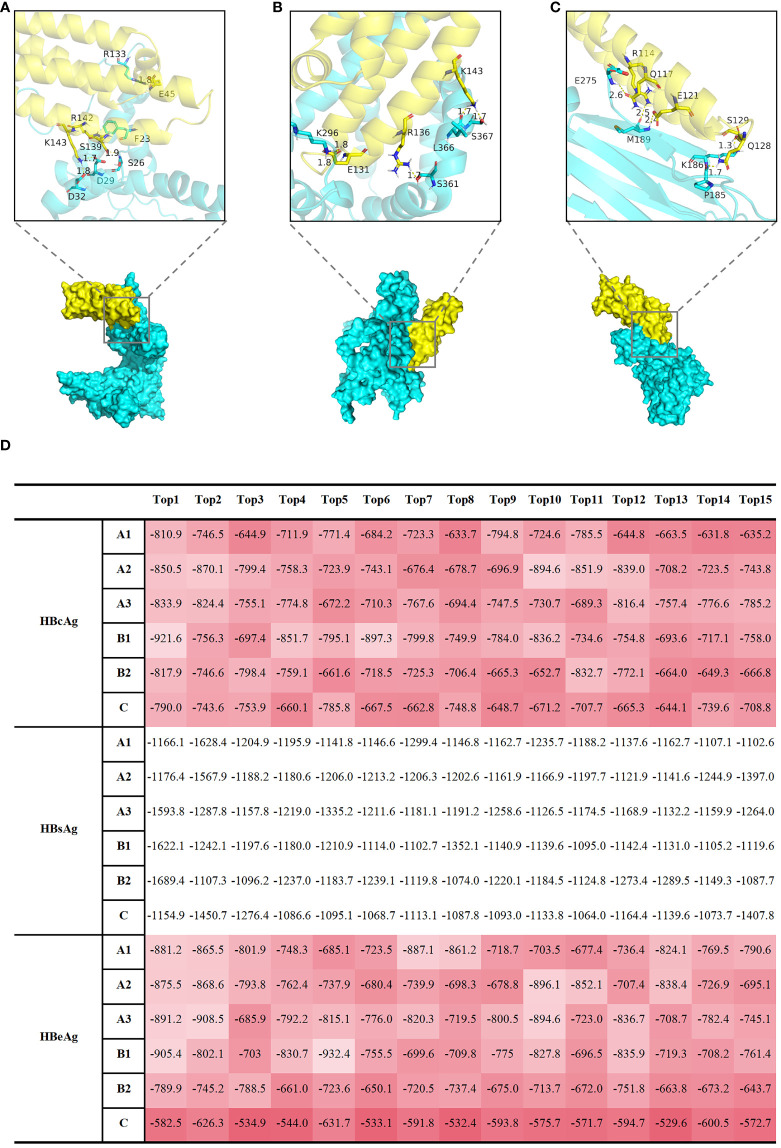
Computational docking analyses were performed to examine the interactions between HBcAg, HBsAg, and HBeAg proteins with APOE molecules, shedding light on potential molecular associations. Display of docking results between representative HBcAg (protein ID, Q9E6S6) **(A)**, HBsAg (protein ID, Q9E6S4) **(B)**, HBeAg (protein ID, P0C6H2) **(C)** proteins, and APOE (protein ID, P02649) molecules. The protein IDs were obtained from UniProt (https://www.uniprot.org/). The yellow protein represents APOE, and the Cyan protein represents HBV. Local graphs depict the Top 5 polar bonds with the closest distances in the docking results. Red denotes oxygen (O) atoms, and blue denotes nitrogen (N) atoms. Numbers indicate the distances between atoms forming polar bonds (unit: angstroms, Å). **(D)** The table displays the top 15 results of the 'balance' calculation weight scores, obtained through the Cluspro2 online analysis platform using the interface root mean square deviation (IRMSD) method. A1, A2, A3, B1, B2, C represent different HBV subtypes, each uniquely identified by specific protein IDs obtained from UniProt (https://www.uniprot.org/). The protein IDs for HBeAg are as follows: Q91C37 (A1 subgenotype), P0C692 (A2 subgenotype), Q4R1S0 (A3 subgenotype), P0C699 (B1 subgenotype), P0C6G7 (B2 subgenotype), P0C6H2 (C subgenotype); The protein IDs for HBsAg are as follows: P31873 (A1 subgenotype), O91534 (A2 subgenotype), Q4R1S6 (A3 subgenotype), Q9QBF0 (B1 subgenotype), Q9QAB7 (B2 subgenotype), Q9E6S4 (C subgenotype); The protein IDs for HBcAg are as follows: I7JHV6 (A1 subgenotype), P0C696 (A2 subgenotype), P0C697 (A3 subgenotype), P0C677 (B1 subgenotype), Q9QAB9 (B2 subgenotype), Q9E6S6 (C subgenotype); The protein IDs for AOPE is P02649. A higher value, represented by a darker red shade in **(D)**, corresponds to a stronger polar bond energy, suggesting a more favorable and robust binding between the two proteins.

However, echoing Cheng Y’s remarks, a fundamental question remains unresolved: How does HBV interact with Apolipoprotein E (ApoE)-rich lipoproteins (21)? Given the high efficacy of HBV in exploiting macrophages for trans-infection of hepatocytes, we posit that HBV may engage in a molecular interaction with ApoE, which could potentially involve their protein structures. To address this question, we performed docking simulations involving Hepatitis B core antigen (HBcAg) (**Figure 2A**), Hepatitis B surface antigen (HBsAg) (**Figure 2B**), Hepatitis B e antigen (HBeAg) (**Figure 2C**) proteins, and ApoE molecules. The top 15 weighted scores of the “balance” calculation, calculated from the interface root mean square deviation (IRMSD) method by the Cluspro2 online analysis platform, are normalized and presented as a heatmap (**Figure 2D**). In contrast to HBsAg, HBcAg, and HBeAg have a more robust binding with ApoE. This kind of conjunction could happen in HBV-uptaking macrophages.

### 2.2 Altered lipid metabolism in CHB livers induces hepatocyte damage by activating DAMPs and PAMPs

As depicted in **Figure 1**, lipid metabolism is altered from the beginning of HBV entry. However, hepatocytes damage with the abnormal elevation of alanine transaminase (ALT) only happened during hepatitis phase of CHB infection. As shown in the study by Allen et al., treatment with BAs did not lead to increased caspase 3 activity in mouse hepatocytes or the release of ALT into the culture medium. This compelling observation strongly supports the notion that BAs do not directly induce apoptosis or necrosis in hepatocytes (22, 23). Furthermore, early growth response protein 1 (Egr1), a critical regulator of various genes, particularly those involved in pro-inflammatory cytokine production and pathways associated with pathogen-associated molecular patterns (PAMPs), was found to be upregulated (22). Additional evidence indicates that BAs need to enter and accumulate within hepatocytes to stimulate cytokines expression, and this effect is not mediated by cytokines membrane receptors (24, 25). Taken together, these findings, observed in both humans and mice, support the hypothesis that when BAs accumulate within hepatocytes, they initiate liver injury by triggering an inflammatory response, leading to the production of pathophysiologically relevant concentrations of chemokines and adhesion molecules, including Monocyte Chemoattractant Protein-1 (MCP-1/Ccl2), Macrophage inflammatory protein-2 (MIP-2/Cxcl2), and Intercellular cell adhesion molecule-1 (ICAM-1) (24) of which lay the foundation for the infiltration of monocytes/macrophages.

**Figure 3:**
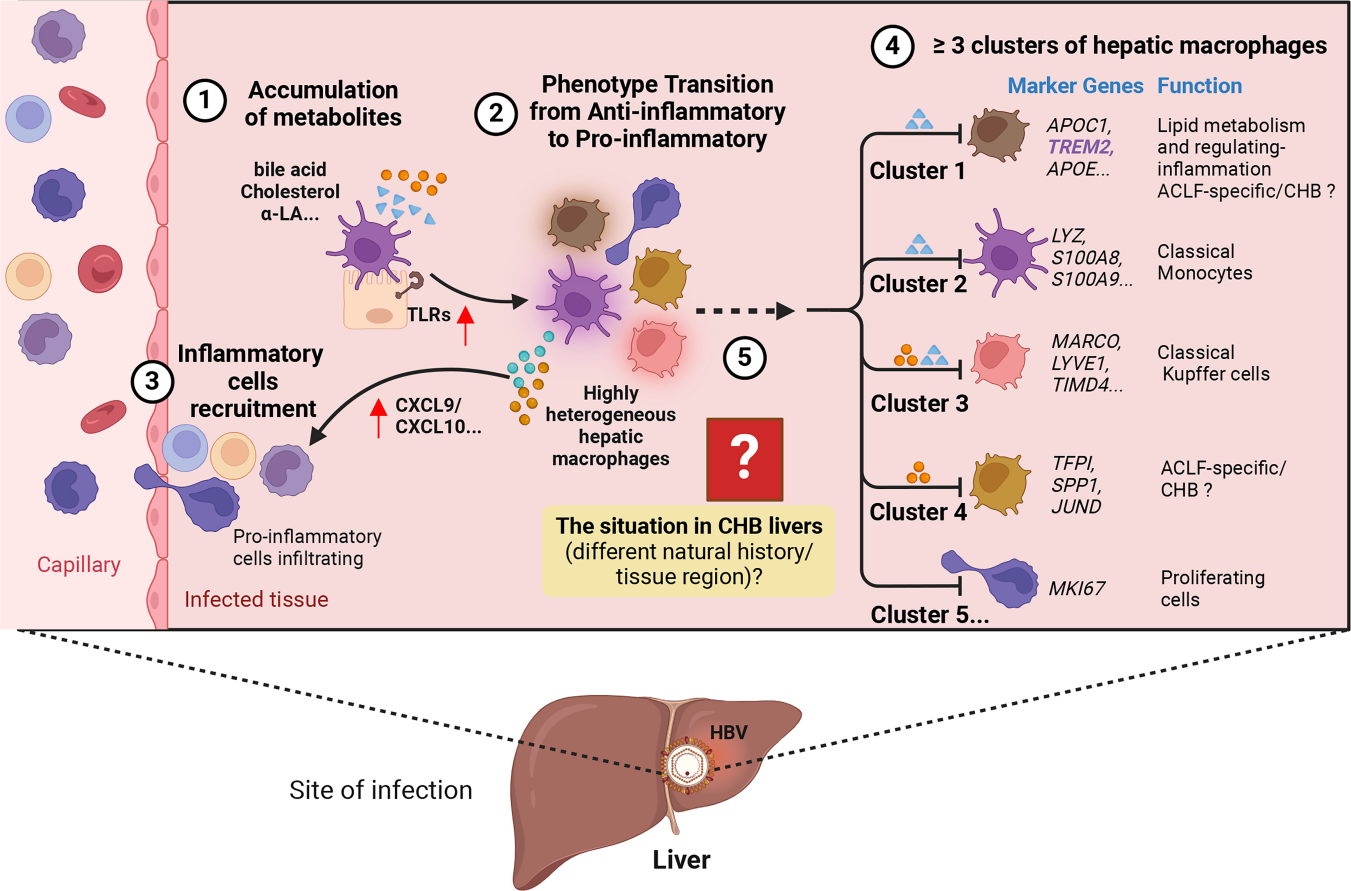
Hypothesis on the process of how HBV induces liver injury. ① Accumulation of metabolites, including bile acids and cholesterol, in the liver. ② Metabolism alteration promotes the expression of pro-inflammatory signaling like Toll-like receptors (TLRs) in hepatocytes, leading to the transition of highly heterogeneous hepatic macrophages into a pro-inflammatory phenotype characterized by increased expression of pro-inflammatory cytokines and activation of specific signaling pathways. ③ More inflammatory cells are recruited into the liver through the release of chemokines like CXCL9/10. ④ Within the highly heterogeneous hepatic macrophages, the presence of at least five potential clusters can be identified in both physiological and pathological states of the liver (e.g., Acute-on-Chronic Liver Failure, ACLF; Chronic hepatitis B, CHB…). Each cluster is associated with specific marker genes and potential functions. ⑤ The phenotypic and functional transitions may exhibit variation across different stages of CHB and diverse liver regions. The figure was created by BioRender (BioRender.com).

Moreover, once these BAs accumulate within the cell, they trigger endoplasmic reticulum (ER) stress and induce mitochondrial damage (25–27). The detection of mitochondrial damage in hepatocytes treated with BAs implies that these damaged mitochondria might release damage-associated molecular patterns (DAMPs), subsequently activating PAMPs, such as TLRs. Notably, TLR9 is a receptor residing within the ER and endosomes, serving as an intracellular DNA sensor. Previous research has shown that mitochondrial DNA can activate TLR9 and stimulate the expression of inflammatory cytokines (27). The involvement of TLR9 is further substantiated by observations that bile acid-induced cytokines responses are diminished in mouse hepatocytes when mitochondria are protected by agents such as cyclosporine A (27). Based on this evidence, we endorse the idea that progressive lipid metabolism alteration induced by HBV infection initiates PAMPs and DAMPs, thereby shaping a pro-inflammatory intrahepatic micro-environment, without direct damage to hepatocytes (**Figure 3**).

### 2.3 Characteristics of intrahepatic metabolic changes during CHB: spatial and temporal specificity

With our advancing understanding of liver anatomy and the liver micro-environment, Guilliams M. et al., emphasized that the liver has a dynamic complexity that has not yet been adequately described (28). We now recognize the liver as a complex organ characterized by a large number of microscopic functional units, including hepatic (classic) lobules, portal lobules, and liver acini. These acini can be divided into three zones based on their proximity to portal canals and adjacent central veins, contributing to uneven blood flow in these three zones. Questions about the relationship between liver blood flow and hepatic metabolism were raised by Macdonald AC as early as 1979 (29). In line with earlier perspectives, Esser K, et al., also explored the deposition of HBV in the perisinusoidal space of Disse, where HBV can bind to its receptor on hepatocytes or even be directly transported to hepatocytes by disrupting the reverse cholesterol transport (20). The precise metabolic alterations induced by HBV in different regions of the liver, various cell types, and at different disease stages remain largely unknown.

Most recently, Li J. et al., conducted an analysis of PBMC transcriptomics from patients in five distinct disease stages, including acute-on-chronic liver failure (ACLF), acute-on-chronic hepatic dysfunction (ACHD), liver cirrhosis (LC), CHB, and normal controls (NC). Their study aimed to demonstrate the presence of immune-metabolism disorders during the development of HBV-ACLF. Notably, they reported increased expression of metabolic genes related to lipid metabolism, fatty acid metabolism, oxygen homeostasis, and autophagy in the PBMC transcriptomics of CHB patients compared to healthy controls (HC) (13). Subsequently, Peng B. et al., characterized the immune micro-environment in the livers of patients with ACLF and also investigated the role of lipid metabolism. They employed single-cell RNA-sequencing (scRNA-seq) to examine liver non-parenchymal cells (NPCs) and PBMCs from healthy controls, cirrhosis patients, and ACLF patients. It’s important to note that well-defined natural history disease stages of CHB patients were not included for further comparison (14).

In 2021, Sun Z. et al., conducted a study comparing serum BA profiles among different groups: CHB patients with normal ALT (CHB-NALT), CHB patients with abnormal ALT (CHB-AALT), and healthy controls (HCs). They employed Ultra-high-performance liquid-chromatography and analyzed transcriptomic data of hepatic gene expression. The findings revealed a significantly higher percentage of conjugated BAs and primary BAs in CHB patients, even in the absence of apparent liver injury. Moreover, CHB-AALT group exhibited higher levels of serum BA species, including glycolithocholic acid (GLCA), taurochenodeoxycholic acid (TDCA), taurolithocholic acid (TLCA), and taurochenodeoxycholic acid (TUCDA) compared to CHB-NALT (30). Clearly, there is a distinct metabolic fingerprint between the inflammatory (hepatitis) and non-inflammatory (non-hepatitis) phases. However, the specific metabolic changes that occur at each of the four natural infection history stages, and the factors that determine the transition between these stages, remain largely unknown.

Despite the rapid advancements in omics-sequencing methods, including spatial transcriptomics and spatial proteomics, there is still a lack of evidence regarding the spatial (tissue/cell-specific) and temporal (different disease stages) metabolic changes within the liver tissues in CHB.

## 3 Limitations of current studies on the mechanisms of HBV-mediated metabolic changes: are hepatocytes the sole targets?

Current studies suggest that hepatocytes are the primary targets of HBV-induced intrahepatic metabolic changes. Are hepatocytes the only targets of HBV-induced intrahepatic metabolic changes? Shin HJ. et al., demonstrated that the HBx promotes gluconeogenesis through the nitric oxide (NO)/c-Jun N-terminal kinases (JNK) pathway (31). Li H. et al., verified that HBV upregulates glycolysis (32). In addition, Liu B. et al., found that HBx-mediated NF-E2-related factor 2 (Nrf2) activation promotes the pentose phosphate pathway (PPP) by stimulating glucose-6-phosphate dehydrogenase (G6PD) expression (33). Furthermore, accumulating evidence from studies using HBV-replicating cell lines and mouse models have shown that HBV can promote fatty acid synthesis through various mechanisms, and is a potential trigger of liver steatosis (6–8, 24, 33–36). Additionally, HBV infection also impacts other hepatic metabolic signaling pathways, such as nucleic acid metabolism and vitamin metabolism (37, 38).

Notably, the identification of the bona fide receptor for HBV, confirmed through stable HBV infection models and human hepatocytes, has revealed that the viral entry mechanisms for HBV (19). However, it remains unclear whether and what extent HBV can influence cellular metabolism via NTCP. Oehler N. et al., subsequently demonstrate that the binding of HBV to NTCP limits its function, subsequently promoting compensatory BA synthesis and cholesterol provision (39). Patman G. et al., further support that HBV infection alters bile acid metabolism (40).

In other words, traditional studies on how HBV infection affects metabolic changes has predominantly relied on hepatoma cell lines, HBV transgenic mice, or peripheral non-tumor tissues after surgical resection. It’s worth noting that hepatocyte-specific transporters are known to be altered or lost in cell lines, mice models, and primary human hepatocyte cultures (**Table 1**). The narrow tissue and host tropism of HBV has constrained studies on interactions established by HBV in human hepatic niche (41, 42).

**Table 1:** Summary of HBV induced metabolic alterations via cells lines, animal models, and clinical specimens.

Cell lines/Mouse background	Plasmid/viral particle	Clinical Specimens (Types and disease stages)	Multi-Omics sequencing	Specific Metabolic changes	Possible Mechanisms/ Regulating pathways/Determinants	Ref
Cell lines: HepG2, Huh7, HepG2.2.15, HepG3B, HepG2-HBV3, control HepG2-RepSal1; Animal models: HBV (HBV-Tg) or HBx+/– transgenic mice (HBx-Tg) and wild-type (WT) littermates	pGL3B-255, pGL3B-255HNF3βmut, and pGL3B-255C/EBPαmut; pGL3B-255PPARαmut and pGL3B-255mut;	Serum of CHB patients	None	Upregulated lipid metabolism, Lipid accumulation	HBx promotes hepatic lipid accumulation through upregulating FABP1 that involves HNF3β, C/EBPα, and PPARα in the development of HBV-induced NAFLD/FABP1	(6)
Cell lines: HBx-transfected Chang liver cells, HepG2-HBx stable cells Animal models: HBx-transgenic mice	pcDNA3.1-Flag-SREBP1a (human, aa 1–490) and pGL2B-FAS-luc (-250 to +65)	None	None	Hepatic lipid accumulation	HBx Stimulates the Gene Expression and the Transcriptional Activation of C/EBPα and PPARγ; Induces the Expression of Adipogenic and Lipogenic Genes	(7)
Animal models: HBx transgenic/AdHBx-GFP-injected mice	NF-κB-luc; pCMV-HA-HBx; pCMV-Tag 2C, pEGFP-C2, and pGEX-4T-2	None	None	Lipid accumulation, steatosis and apoptosis	HBx-induced NF-κB activation was associated with the induction of steatosis and apoptosis through promotion of TNF-α production, which is determined by a TNFR1-dependent pathway.	(8)
Cell lines: HepG2 and AD293 cells	Ad-HBV	None	None	Cholesterol accumulation	Ad-HBV increases LDLR and HMGCoAr expression, resulting in exacerbated cholesterol accumulation in HepG2 cells, which was mediated via the TLR2 pathway.	(9)
None	None	PBMCs from subjects with HBV-ACLF, ACHD, LC or CHB and NC	mRNA sequencing	Prominent metabolic alterations (including lipid metabolism, fatty acid metabolism, autophagy and oxygen homoeostasis)	Increasing expression of MERTK, SEMA6B and THBS1	(13)
None	None	PBMCs and NPCs from healthy controls, cirrhosis patients and ACLF patients	Single-cell RNA-sequencing of liver NPCs	Upregulated lipid metabolism in ACLF patients	An increase of α-LA and α-LA metabolism and beta oxidation of very long chain fatty acids. Genes related to lipid metabolism, including APOE, APOC1, FABP5 and TREM2, were upregulated along the pseudotemporal trajectory from Mono2 to Mono1	(14)
Cell lines: HepG2, HepG2.2.15, and Huh7 cells	None	Frozen fresh liver or liver cancer tissues; Tumor liver tissues and their peripheral non-tumor tissues after surgical resection were collected from HCC patients with chronic HBV infection	None	Upregulated glucose metabolism	The formation of HBx–p62–Keap1 complex initiated by HBx is essential to HBx-stimulated Nrf2 activation; HBV-stimulated Nrf2 activation and G6PD expression	(33)
Cell lines: Chang liver (A.T.C.C. CCL 13), HepG2, Huh7 and Hep3B	pcDNA3/GST/HBx, pM/HBx (Gal4/HBx) and pVP16/HBx;	None	None	Upregulated hepatic lipid accumulation via fatty acid synthase and lipogenic genes	HBx interacts with LXRα and enhances the binding of LXRα to LXRE, thereby resulting in the up-regulation of SREBP1 and FAS; HBx also augments the ability to recruit ASC2 that control liver lipid metabolic pathways, to the LXRE with LXRα.	(36)
None	None	Serums from treatment-naïve patients with chronic HBV infection	None	Low 25(OH)D3 serum levels	None	(38)
Cell lines: HepG2, Huh-7, SMMC-7721 (SMMC), and Bel-7404	AAV8-HBV	PTHs, PHHs	LC-MS/MS	None	None	(10)
Animal models: HBV-infected chimeric mice (Human hepatocytes were injected into uPA/SCID mice)	None	Human liver tissues were obtained from needle liver biopsy specimens	None	Accumulated lipid and bile acid metabolism	PreS1—which binds and suppresses the activity of the bile acid transporter NTCP—a similar induction of CYP7A1, a transcriptional repressor (SHP) of CYP7A1 is decreased	(40)
None	None	CHB-NALT, CHB-AALT,and HCs	UPLC-MS measurement	Higher Bile Acids for chronic HBV infection	Higher GLCA, TDCA, TLCA and TUCDA levels were found in CHB-AALT patients than CHB-NALT; Gradual increases of conjugated forms of primary BAs, i.e. GCA, TCA, GCDCD, TCDCA were observed in CHB-NALT and CHB-AALT patients	(31)
None	None	ex vivo human liver tissue perfusion model; purified VP comprising a mixture of virions and filamentous subviral particles that express all 3 HBV envelope proteins (S, M, and L) and labelled them with Alexa594	None	HBV hijacks the physiological lipid transport pathways	ApoE Enhances HBV Infection and HBV Localizes to Cellular Compartments That Accumulate Free Cholesterol	(20, 21)

α-LA, α-linolenic acid; APOE, apolipoprotein E; APOC1, apolipoprotein C1; ACHD, acute-on-chronic hepatic dysfunction; ASC2, activating signal co-integrator-2; BA, bile acid; CYP7A1, Cytochrome P450 Family 7 Subfamily A Member 1; C/EBPα, CCAAT enhancer binding protein α; CHB, chronic hepatitis B; CHB-NALT, CHB patients with normal ALT; CHB-AALT, CHB patients with abnormal ALT; CHKA, choline kinase alpha; FABP5, Fatty acid-binding protein 5; GCA, Glycocholic acid; GCDCD, Glycochenodeoxycholic acid; HCs, healty controllers; HCC, Hepatocellular Carcinoma; HBx, hepatitis B virusXprotein; HBV-ACLF, HBV-induced acute-on-chronic liver failure; LXR, liver X receptors; LCs, liver cirrhosis (LC); LC-MS, Liquid Chromatography with tandem mass spectrometry; LXRE, LXR-response element; MerTK, Mer tyrosine kinase; NPCs, non-parenchymal cells; NAFLD, Nonalcoholic fatty liver disease; PBMC, peripheral blood mononuclear cells;PPARα, Peroxisome proliferator-activated receptor; PTHs, Primary Tupaia hepatocytes; PHHs, Primary human hepatocytes; SEMA6B, semaphorin 6B; TDCA, Taurodeoxycholic acid; TLCA, Taurolithocholic Acid; TUCDA,Tauroursodeoxycholic acid; TCA, Taurocholic Acid; TCDCA, Taurochenodeoxych.

## 4 Function of hepatic macrophages niche: shaping intrahepatic micro-environment for immune tolerance or infiltration

### 4.1 Viral replication magnitude: a contradictory determinant of immune tolerance and immune activation

It is known that KCs prefer to reside in the liver, and they are enriched with an array of pattern recognition receptors (PRRs) on cellular surface, including TLRs and nucleotide-binding oligomerization domain-like receptors (NLRs). This localization and receptor expression make them first-line responders to pathogens such as HBV. Liver macrophages are also capable of directly recognizing HBV (43, 44). However, the long-standing debate as to whether HBV induces immune activation or promotes liver tolerance remains unresolved.

Liu J. et al., demonstrated that HBV stimuli induce KC-mediated T cell tolerance through the secretion of IL-10 in a TLR2-dependent manner (45). Li’s research further supported the concept that KCs Support HBV-Mediated CD8+ T cell exhaustion via the TLR2 signaling pathway (46). Suzanne Faure-Dupuy et al., reported that exposure to HBV or HBV-producing cells during differentiation and activation led to distinct responses. Pro-inflammatory macrophages (namely M1-MDMs) secreted lower levels of IL-6 and IL-1β, while anti-inflammatory macrophages (namely M2-MDMs)secreted higher levels of IL-10 when exposed to HBV during activation (47). In conclusion, their findings suggest that KCs play a role in promoting the secretion of anti-inflammatory cytokines such as IL-10 and TGF-β, contributing to transient activation by secreting IL-6, and potentially increasing the expression of inhibitory checkpoint factors (e.g., PD-1/PD-L1) (48). These results raise the possibility that HBV-driven limited activation of hepatic macrophages could be a contributing factor to the establishment and persistence of infections *in vivo*.

However, Cheng X. et al., provided evidence that HBV has evolved to evade the innate immunity of hepatocytes but activates macrophages during infection (49). Cheng’s research underscored that macrophage could detect HBV, but this typically requires exposure to high HBV titers. This observation may help explain the prolonged “window period” observed during acute infection and why HBV tends to establish chronic infections. In the most recent study, it was found that the magnitude of viral replication and the specific anti-viral immune responses should ideally dictate the extent of inflammation. However, it’s important to note that a direct correlation between these factors is not consistently observed in patients with chronic viral hepatitis (50).

In summary, HBV-mediated hepatic macrophages have the potential to induce either an anti-inflammatory or pro-tolerogenic differentiation. This phenotypic/functional switch may either support or inhibit further liver pathogenesis in CHB. Nevertheless, the precise mechanisms responsible for chronic liver inflammation and the key factors influencing the various stages of the disease’s natural history are yet to be fully elucidated (**Figure 3**).

### 4.2 Hepatic macrophage niche are attracted to sites where lipid metabolites are exposed for clearance

Single-cell and spatial transcriptomic technologies have unveiled an underappreciated heterogeneity among liver macrophages, prompting us to reconsider the role of macrophages in liver homeostasis and disease (17, 18). Previous research has categorized five hepatic macrophage clusters with distinct transcriptomes, including the MARCO^hi^ cluster, Trem2^hi^ cluster, S100A8^hi^ cluster, MMP19^hi^ cluster, and MKi67^hi^ cluster in ACLF livers (13). Guilliams M, et al., further provided a more comprehensive depiction of the human liver atlas and identified two subsets of the Trem2^hi^ cluster (mature and immature), which they later named Lipid-associated macrophages (LAMs) (18). Importantly, a notion supported by the induction of LAMs marker genes through the treatment of murine bone marrow macrophages with lipids *in vitro*. Besides, Ramachandran P. et al., demonstrated location of LAMs around the bile ducts in the healthy mouse, human, and macaque liver. However, in the presence of steatosis, LAMs are preferentially recruited to the steatotic regions of the liver (51). Guilliams M’s research suggests that LAMs can be induced by local lipid exposure (18).

Trem2 signaling has been recognized as a crucial sensor of metabolic pathology, responding to disruptions in tissue-level lipid homeostasis and giving rise to a novel and conserved Trem2^+^LAM subset (52–54). In accordance with these studies, Trem2^+^macrophages were found to express lipid metabolism genes marked by *APOC1*, *APOE*, *GPNMB*, and *SPP1*, as well as pro-inflammatory genes including *IL18*, *CCL5*, *CCL18*, among others (55). Additionally, the first identified scar-associated macrophages in liver cirrhosis, known as Trem2^+^CD9^+^macrophages, were found to originate from circulating monocytes and expand in the context of liver fibrosis. These Trem2^+^CD9^+^macrophages displayed pro-fibrogenic effects, contributing to tissue repair following liver injury (55). As metabolic alterations play a pivotal role in reshaping the hepatic micro-environment niche, including the recruitment of Trem2^+^monocytes/macrophages and the activation of Trem2^+^monocytes/macrophages toward a pro-inflammatory state, the precise phenotype and functional changes in the micro-environment of CHB livers, as well as variations across disease stages, remain unclear.

In a recent study, Dib L. et al., demonstrated that LAMs undergo a transition to an inflammatory state in human atherosclerosis. This transition revealed the presence of PLIN2^hi^/TREM1^hi^ macrophages, which are associated with TLR-dependent inflammation and linked to cerebrovascular events (53). Peng B. et al., shed light on the immune-suppressive role of Trem2^+^monocytes/macrophages in the context of late-stage ACLF livers (14). Notably, their study provided further evidence that the accumulation of α-linolenic acid (α-LA) could enhance the expression of Trem2 on monocytes/macrophages in an inflammatory environment. This phenomenon was demonstrated through *in vitro* experiments involving LPS and α-LA stimulation on CD14^+^cells, suggesting that increased levels of unsaturated free fatty acids (FFAs) and disturbances in α-LA metabolism may serve as a bridge facilitating the differentiation and activation of Trem2^+^monocytes/macrophages in ACLF livers (14).

**Figure 4:**
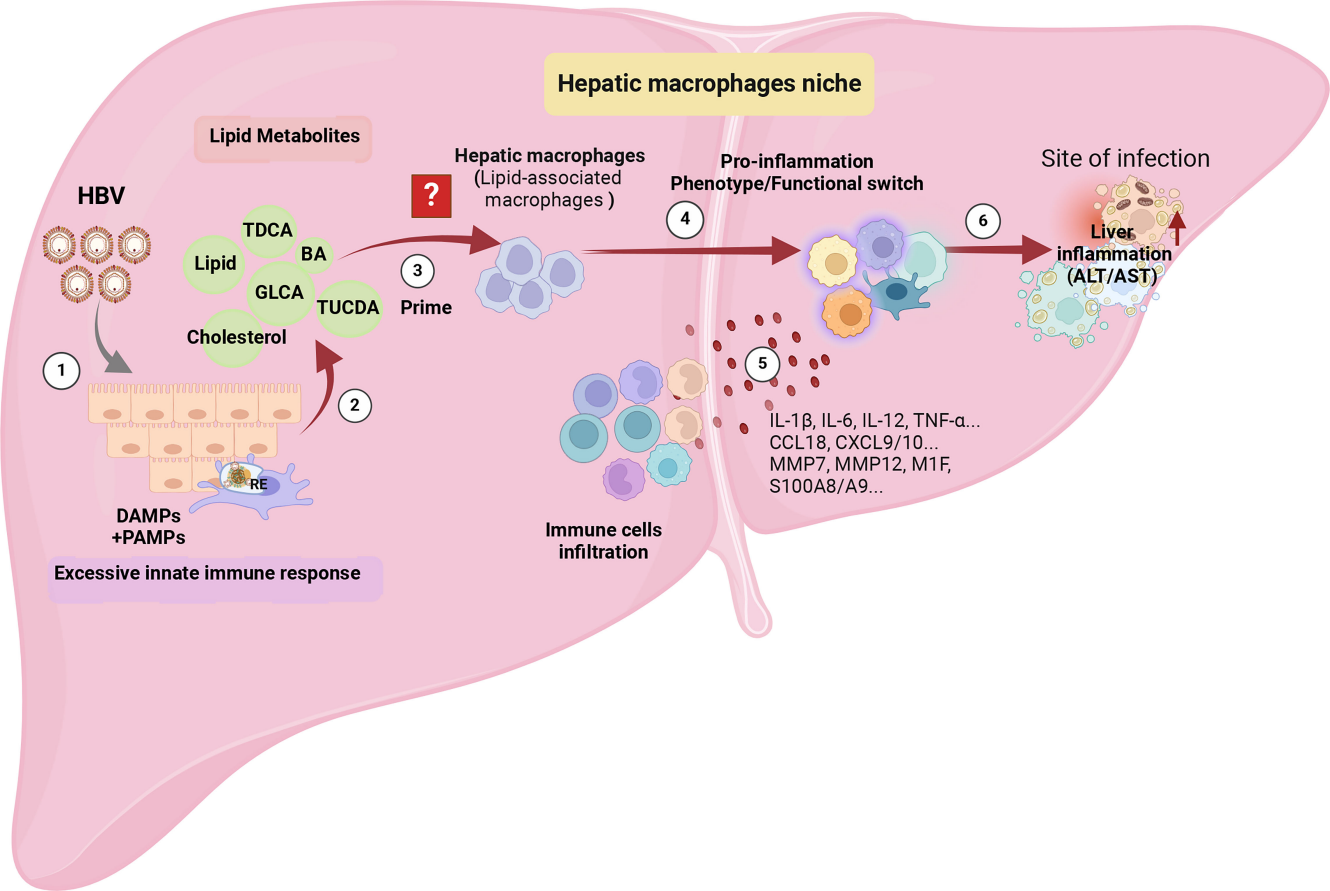
The potential immunopathology of how HBV shapes hepatic macrophage niches, ultimately leading to liver inflammation. ① Persistent HBV stimulation induces abnormal lipid metabolism, characterized by the accumulation of metabolites such as bile acids and cholesterol in the liver microenvironment. ② The aberrant lipid metabolism triggers an innate immune response by releasing PAMPs, such as the up-regulation of TLRs’ expression in hepatocytes, and DAMPs released by damaged hepatocyte mitochondria. ③ The accumulation of lipids recruits a specific cluster of macrophages, possibly Lipid-Associated Macrophages (LAMs), identified by the expression of Trem2, triggering a transition into a pro-inflammatory phenotype. ④ The activated LAMs exhibit a distinctive transcriptomic profile with elevated expression of IL-1β, IL-6, IL-12, TNF-α, CCL18, CXCL9/10, MMP7, MMP12, M1F, S100A8/A9, leading to the recruitment of more inflammatory cells. ⑤ Deepening liver inflammation occurs as a result of increased infiltration of inflammatory cells into the liver. The figure was created by BioRender (BioRender.com).

In the context of CHB, the binding of HBV pre-S1 to NTCP hinders bile acid uptake and compensatory upregulates the expression of cholesterol synthesis genes, including 3-hydroxy-3-methylglutaryl-coenzyme A (HMG-CoA) reductase and the LDL receptor. Consequently, this process is associated with the promotion of TLR signaling and inflammasome activation in hepatic macrophages (12, 13), suggesting it’s bridge role between enhancing lipid metabolites and pro-inflammation. However, HBV exploits NTCP in hepatocytes, disrupting the cholesterol transport pathway between macrophages and their target hepatocytes (10). Whether the specific type of hepatic macrophages involved, such as whether it is LAMs, known to be activated by local lipid exposure, and their role in eliminating excess metabolites and metabolic intermediates, triggering phenotype and functional reprogramming of the hepatic infiltrating microenvironment, remains unknown and is worthy of attention (**Figure 4**).

### 4.3 Pro-inflammatory hepatic macrophages, orchestrating increased liver infiltration and exacerbating liver inflammation

Traditional research categorized liver macrophages into “M1” or “M2” subsets, with a focus on their phenotype shifts and functional changes. M1 macrophages are associated with a pro-inflammatory effect, producing cytokines such as tumor necrosis factor (TNF)-α, IL-1β, and reactive oxygen species (ROS), which contribute to liver inflammation and injury as the disease progresses. In contrast, M2 macrophages exhibit an anti-inflammatory phenotype. It’s important to note that liver macrophages niche is highly plastic and can rapidly transition from a homeostatic state to a pro-inflammatory state in response to viral infection (56, 57).

Accordingly, we hypotheses that, the prolonged disruption in lipid and cholesterol efflux between hepatic macrophages and hepatocytes during the immune tolerance stage of CHB patients leads to the accumulation of excessive cholesterol and lipid metabolites. This, in turn, exacerbates pro-inflammatory signaling through both PAMPs and DAMPs within host cells. Consequently, this process reshapes the macrophage niche, inducing a transition in the immune micro-environment from macrophage-mediated tolerance to immune activation (58).

In Do TH’s research, Trem2^+^macrophages display heightened lipid metabolism profiles alongside pro-inflammatory profiles, resulting in the up regulation of pro-inflammatory chemokines, cytokines, MMPs, and S100 proteins to further recruit and activate immune cells (55). In Dib L’s research, once the macrophages shift into a pro-inflammatory phenotype (PLIN2^hi^/Trem1^hi^), they exhibit a unique chemokine signature with transcripts for pro-atherogenic CCR2 ligands (CCL2 and CCL7), which play a non-redundant role in atherogenesis and monocyte recruitment (51). Aberrant immune metabolism in the liver of CHB leads to pro-inflammatory macrophage activation. This, in turn, sustains the production of pro-inflammatory cytokines, including Chemokine (C-X-C motif) ligand 9/10 (CXCL-9/CXCL-10), and others, which are essential for recruiting lymphocytes and monocytes/macrophages to the liver tissue (15, 58). These studies provide further support for the concept that pro-inflammatory hepatic macrophages play a central role in enhancing liver infiltration and exacerbating liver inflammation (**Figure 4**) (19).

## 5 Future perspectives

CHB is characterized by distinct and dynamic disease progression stages, yet the mechanism governing host immune responses, particularly within the liver, and the development of liver pathology throughout these stages remain elusive. In this context, we highlight the pivotal role of hepatic macrophages in both recognizing HBV through innate immunity and evading HBV immune surveillance through various mechanisms. However, the specific trigger responsible for shifting the immune tolerance state of the CHB-infected liver towards an immune-activated state, ultimately resulting in liver inflammation and liver injury, remains unidentified.

As the body of evidence regarding the involvement of HBV in metabolic processes grows, it becomes increasingly clear that HBV employs a dual strategy for entering target cells (10, 13, 21). Besides, our review initially emphasizes the significant role of HBV-induced metabolic changes in hepatic macrophages as a key factor in the transition from viral infection to immune activation, ultimately leading to liver inflammation. We propose that metabolic alterations in specific hepatic macrophages subsets are crucial for CHB liver inflammation and disease progression. Our concept presents new potential intervention strategies for achieving a functional cure in CHB: early blockade of HBV-induced metabolic changes and inflammation; modulation of the pro-inflammatory phenotype of liver-resident macrophages by targeting specific markers or genes; and targeted metabolic therapy with antiviral and anti-inflammatory effects.

However, current studies on the mechanisms of HBV-mediated metabolic changes and intrahepatic inflammation have been conducted either in hepatoma cell lines like HepG2 and Huh7, HBV transgenic mice, or peripheral non-tumor tissues after surgical resection. These *in vitro* experiments are insufficient to accurately reflect the levels of HBV-mediated metabolic and inflammatory changes (8, 24, 31–33, 36–38). Although the rapid advancement of omics technologies, multi-omics studies on clinical liver biopsy samples have compensated for the lack of observations on real clinical samples, existing research still lacks monitoring of samples from different stages of CHB’s natural history, as well as pathological and omics-level monitoring of different spatial or anatomical regions (13, 14, 17, 18, 51–55).

Our latest insights underscore that HBV induces significant alterations in metabolic pathways, particularly within the interplay between hepatocytes and the hepatic macrophage efflux system during HBV infection (13, 21). This dynamic interaction serves as a critical link connecting HBV infection and pro-inflammatory activation. While this hypothesis is promising, it necessitates further experimental validation. Several unresolved issues warrant additional emphasis: 1) The specific metabolic profiles for distinct cell clusters, particularly hepatocytes and macrophages at different stages of CHB, remain un-characterized. 2) The degree of liver injury and the extent of changes in key cellular metabolic factors have not yet be established. 3) In-depth understanding of spatial zonation into CHB liver by multi-omics and subsequent validations are required.

Therefore, future studies should prioritize diverse cohorts representing different stages of CHB natural history. Combining multi-omics sequencing technologies to analyze these abundant clinical samples is essential for a comprehensive understanding and control of changes at the genomic, proteomic, metabolomic, and other levels. Bioinformatic analyses should focus on characterizing cell cluster-specific metabolic profiles within the liver and examining correlations between metabolic changes and key biomarkers associated with immune activation or liver injury. Comprehensive omics-sequence analyses and subsequent research are crucial to substantiate these findings, aiming to elucidate the spatial and temporal dynamics of intrahepatic metabolites and immune-pathological profiles. Additionally, effective animal models of inflammation progression and *in vitro* experiments are needed to complement omics findings and confirm our conclusion: the potential role of hepatic macrophages in activating metabolic changes and inflammatory responses that ultimately result in liver injury.

## Author contributions

JW: Conceptualization, Writing – original draft, Writing – review & editing. HL: Writing – review & editing. QL: Conceptualization, Supervision, Visualization, Writing – original draft, Writing – review & editing.

## Glossary

**Table:**

α-LA	α-linolenic acid
APOE	apolipoprotein E
APOC1	apolipoprotein C1
ACHD	acute-on-chronic hepatic dysfunction
ASC2	activating signal co-integrator-2
ALT	alanine transaminase
BA	bile acid
CYP7A1	Cytochrome P450 Family 7 Subfamily A Member 1
C/EBPα	CCAAT enhancer binding protein α
CHB	chronic hepatitis B
CHB-NALT	CHB patients with normal ALT
CHB-AALT	CHB patients with abnormal ALT
CHKA	choline kinase alpha
DDB1	damage binding protein 1
Egr1	early growth response protein 1
ER	endoplasmic reticulum
FABP5	Fatty acid-binding protein 5
FFAs	free fatty acids
FXR	Farnesoid X receptor
G6Pase	glucose-6-phosphatase
GFAT1	glutamine-fructose-6-phosphate amidotransferase 1
GCA	Glycocholic acid
GCDCD	glycochenodeoxycholic acid
GLCA	glycolithocholic acid
NO	nitric oxide
JNK	c-Jun N-terminal kinases pathway
GnT-III	N-acetylglucosaminyltransferase-III
MASLD	Metabolic Dysfunction-Associated Fatty Liver Disease
HC	healthy controller
HCC	Hepatocellular Carcinoma
HBx	HBV X protein
HBV-ACLF	HBV-induced acute-on-chronic liver failure
HBcAg	Hepatitis B core antigen
HBsAg	Hepatitis B surface antigen
HBeAg	Hepatitis B e antigen
HNF-4α	nuclear factor-4 alpha
HMGCR	3-hydroxy-3-methylglutaryl-CoA reductase
KCs	Kupffer cells
LXR	liver X receptors
LCs	liver cirrhosis
LC-MS-MS	Liquid Chromatography with tandem mass spectrometry
LXRE	LXR-response element
LAMs	Lipid-Associated Macrophages
LRH-1	liver receptor homolog-1
LDLR	low-density lipoprotein receptor
MerTK	Mer tyrosine kinase
MDMs	monocyte-derived macrophages
NPCs	non-parenchymal cells
NCs	normal controls
NTCP	sodium taurocholate co-transporting polypeptide
NLRs	nucleotide-binding oligomerization domain-like receptors
NAFLD	Nonalcoholic fatty liver disease
PBMC	peripheral blood mononuclear cells
PEPCK	Phosphoenolpyruvate Carboxykinase
PRRs	pattern recognition receptors
PPARα	Peroxisome proliferator-activated receptor α
PTHs	Primary Tupaia hepatocytes
PHHs	Primary human hepatocytes
AKT	Phosphoinositide 3-kinase (PI3K)/protein kinase B
ROS	reactive oxygen species
SHP	small heterodimer protein
SEMA6B	semaphorin 6B
SREBP1	Sterol Regulatory Element-binding Protein-1
scRNA-seq	single-cell RNA-sequencing
TDCA	Taurodeoxycholic acid
TLCA	Taurolithocholic Acid
TLRs	Toll-like receptors
TUCDA	Tauroursodeoxycholic acid
TCA	Taurocholic Acid
TCDCA	Taurochenodeoxych
TNF-α	tumor necrosis factor
UPLS-MS	Ultra-performance liquid chromatography-mass spectrometry
VLDL	very low-density lipoprotein
